# Altered placental expression of PAPPA2 does not affect birth weight in mice

**DOI:** 10.1186/1477-7827-8-90

**Published:** 2010-07-20

**Authors:** Pamela K Wagner, Julian K Christians

**Affiliations:** 1 Simon Fraser University, Biological Sciences, 8888 University Drive, Burnaby, BC, V5A 1S6, Canada

## Abstract

**Background:**

Pregnancy-associated plasma protein A2 (PAPPA2) is an insulin-like growth factor binding protein (IGFBP) protease expressed in the placenta and upregulated in pregnancies complicated by pre-eclampsia. The mechanism linking PAPPA2 expression and pre-eclampsia and the consequences of altered PAPPA2 expression remain unknown. We previously identified PAPPA2 as a candidate gene for a quantitative trait locus (QTL) affecting growth in mice and in the present study examined whether this QTL affects placental PAPPA2 expression and, in turn, placental or embryonic growth.

**Methods:**

Using a line of mice that are genetically homogenous apart from a 1 megabase QTL region containing the PAPPA2 gene, we bred mice homozygous for alternate QTL genotypes and collected and weighed placentae and embryos at E12.5. We used quantitative RT-PCR to measure the mRNA levels of PAPPA2, as well as mRNA levels of IGFBP-5 (PAPPA2's substrate), and PAPPA (a closely related IGFBP protease) to examine potential feedback and compensation effects. Western blotting was used to quantify PAPPA2 protein. Birth weight was measured in pregnancies allowed to proceed to parturition.

**Results:**

PAPPA2 mRNA and protein expression levels in the placenta differed by a factor of 2.5 between genotypes, but we did not find a significant difference between genotypes in embryonic PAPPA2 mRNA levels. Placental IGFBP-5 and PAPPA mRNA expression levels were not altered in response to PAPPA2 levels, and we could not detect IGFBP-5 protein in the placenta by Western blotting. The observed difference in placental PAPPA2 expression had no significant effect on placental or embryonic mass at mid-gestation, birth weight or litter size.

**Conclusions:**

Despite a significant difference between genotypes in placental PAPPA2 expression similar in magnitude to the difference between pre-eclamptic and normal placentae previously reported, we observed no difference in embryonic, placental or birth weight. Our results suggest that elevated PAPPA2 levels are a consequence, rather than a cause, of pregnancy complications.

## Background

Insulin-like growth factors (IGFs) play a pivotal role in fetal and placental development [1]. The bioavailability of the IGFs is regulated by six IGF binding proteins (IGFBPs), and release of IGFs and subsequent IGF signalling is achieved primarily through cleavage of the IGFBPs by proteases [2-4]. Pregnancy-associated plasma protein A (PAPPA) is an IGFBP protease expressed at high levels by the placenta, and is the main enzyme responsible for the cleavage of IGFBP-4 in this tissue [5,6]. Circulating levels of PAPPA are elevated dramatically during pregnancy, and prenatal testing for low levels of PAPPA in maternal serum is currently used to screen for Down's syndrome and other aneuploidies [7,8]. Abnormally low levels of PAPPA are also associated with preterm delivery [9], pre-eclampsia [9,10], intrauterine growth restriction (IUGR) [11,12], and other adverse outcomes such as stillbirth [9].

Pregnancy-associated plasma protein A2 (PAPPA2), also known as pappalysin-2 and PAPP-E [13], is another IGFBP protease produced at high levels by the placenta that has received much less study. PAPPA2 is 45% identical to PAPPA at the amino acid level [14] and, in addition to similarity in sequence, PAPPA and PAPPA2 have similar substrates: PAPPA cleaves IGFBP-4 and perhaps IGFBP-2 and IGFBP-5 [15], while PAPPA2 is only known to cleave IGFBP-5 [14]. Recently, PAPPA2 has been shown to be responsible for the IGFBP-5 proteolytic activity of pregnancy plasma, resulting in increased IGF bioavailability [16]. Furthermore, four other studies have found PAPPA2 to be upregulated in placentae from pregnancies complicated by pre-eclampsia and a pre-eclampsia-like syndrome, HELLP (Hemolytic anemia, Elevated Liver enzymes, and Low Platelet count) [17-20]. However, it is not clear whether PAPPA2 expression is upregulated to compensate for abnormal placentation or whether elevated PAPPA2 levels cause abnormal placental development, leading to hypertensive disorders [21]. Elevated PAPPA2 levels might be expected to increase IGFBP-5 proteolysis and so increase IGF availability and placental development. However, Winn *et al*. [20] suggested that increased PAPPA2 levels could reduce stimulatory effects of IGFBP-5, and so reduce cytotrophoblast invasion of the decidua, an important step in placental development. IGFBP signalling is complex, and IGFBP-5 has been shown to have stimulatory and IGF-independent roles [22]. An animal model is therefore needed to elucidate the functional role of PAPPA2 during pregnancy. Recent work has shown that PAPPA2 is expressed at high levels in the murine placenta and that it is expressed in along the fetal-maternal interface of the placenta in both mouse and human [23], suggesting that the mouse may be a suitable model for studying the placental roles of PAPPA2.

In light of the recent work that found PAPPA2 to be upregulated in human pre-eclamptic placentae [17-20], we sought to investigate the phenotypic consequences of altered placental PAPPA2 expression in mice. To do this, we examined natural variation in the PAPPA2 gene that has previously been associated with postnatal growth in mice: quantitative trait locus (QTL) mapping revealed that a chromosomal region containing the PAPPA2 gene affects body size [24]. The QTL region contains only four genes, including PAPPA2, and affects the circulating levels of IGFBP-5, PAPPA2's substrate [24], suggesting that PAPPA2 is a strong candidate gene for this QTL.

The goal of the present study was to examine whether this QTL affects placental PAPPA2 expression in mice and, if so, whether altered expression of this gene has phenotypic consequences. Pre-eclampsia results from abnormal placentation combined with risk factors that predispose the mother to hypertension [25-27]. Since PAPPA2 is expressed at high levels in the placenta, we predicted that altered PAPPA2 expression might influence placental development, but not necessarily the risk of hypertension. Therefore, we focused on placental weight, embryonic weight and birth weight as phenotypic outcomes since intrauterine growth restriction is also caused by abnormal placentation and is often associated with pre-eclampsia [26,28]. Furthermore, Nishizawa *et al. *[18] found negative correlations between birth weight and placental weight and circulating PAPPA2 levels when combining data from normal and pre-eclamptic pregnancies. We examined the effects of variation in the QTL region using a line of mice that is genetically homogenous but segregates for a 1 megabase (MB) region containing the QTL [24]. Although altered placental expression of PAPPA2 is not expected to be the mechanism underlying the postnatal growth QTL described previously, variation in the PAPPA2 gene could have pleiotropic effects on prenatal and postnatal growth.

## Methods

### Mice and sample collection

In previous work, we introgressed the C57BL/6 allele of a chromosome 1 QTL into the DBA/2 background to produce a line segregating for an approximately 1-MB region containing the QTL [24]. This line is currently at backcross generation 12. The mice used in this study were all obtained from matings between mice heterozygous for the QTL region, i.e., the grandparents of all of the embryos and pups studied were of the same genotype. These matings produced litters in which offspring varied for QTL genotype, and we paired mice of the same homozygous QTL genotype (i.e. homozygous C57BL/6 or homozygous DBA/2) to produce placentae and embryos homozygous for the alternate genotypes. Females were checked for seminal plugs the morning following pairing, and plugged females were euthanized with CO_2 _twelve days later (embryonic day 12.5). Since the abnormal placentation that is thought to cause pre-eclampsia and intrauterine growth restriction occurs early in pregnancy [26], we wanted to sample placentae as early in pregnancy as possible, while ensuring that individual placentae and embryos would be sufficiently large for mRNA extraction. We therefore chose to collect females at embryonic day 12.5. Preliminary data indicated that placental PAPPA2 mRNA levels were similar at E10.5, E12.5 and E18.5 (data not shown), and placental PAPPA2 protein levels have been found to be similar from E11.5 to E15.5 by immunohistochemistry [23].

Placentae and embryos were dissected while immersed in 10× PBS treated with DEPC, then weighed. The number of embryos and spontaneous abortions were counted; aborted embryos were characterized by a small region of uterine horn containing dark tissue, without any sign of placenta or embryo. Half of the placenta and embryo samples were stored in RNAlater^® ^(Ambion, Foster City, CA) for quantification of mRNA levels by quantitative real-time PCR (qRT-PCR), and the other half were frozen at -80°C for quantification of protein levels by Western blotting.

To determine birth weight, females were not checked for plugs, but were instead left with males, checked daily three to four and a half hours after the lights were turned on, and new pups were weighed. In addition to the measurement of birth weight in pups from the homozygous matings described above, we also weighed pups from heterozygous matings, where genotype varied within litters. We anaesthetized the newborn offspring of heterozygous pairings using isofluorane, and collected 2 mm of tail tissue for genotyping. We genotyped pups by PCR using two microsatellite markers that delimit the QTL region (D1Icp1 primers: CTTTGACAGGAGTGGCTGAT and TTGAAGGCTAGTCTCTCTACTGG; D1Icp9 primers: TTCCAAATCCTGCCACTATC and CAAACATCCAGTCCTTCTCC). All work was carried out in accordance with the guidelines of the Canadian Council on Animal Care and approved by the SFU Animal Care committee (protocol 771B-05).

### RNA isolation and qRT-PCR

Placentae and embryos were homogenized at room temperature in 600 μL of buffer RLT (Qiagen, Ontario, Canada) using pestles. The samples were further homogenized using Qiashredders (Qiagen, Ontario, Canada) and total RNA was extracted using the RNeasy Mini kit (Qiagen, Ontario, Canada) according to the manufacturer's instructions. RNA concentration was determined using a Nanodrop spectrophotometer (Thermo Fischer Scientific Inc. Waltham, MA), and each sample was diluted to a final concentration of 50 ng/μL. Reference samples were prepared by combining aliquots of placental or embryonic RNA extracts, and separate reference samples were used for placenta and embryo. These reference samples were included in every assay to account for variation between assays. Quantitative real time (qRT) PCR was performed to compare levels of PAPPA2 mRNA between the two different genotypes. Where significant differences in PAPPA2 levels were observed, we also compared mRNA levels of IGFBP-5, the substrate of PAPPA2, and PAPPA, a closely related protease, to test for feedback or compensation effects. Levels of β-actin were also measured and used as a reference to standardize the values of the other genes (see Table 1 for primer and probe sequences). The qScript 1-step qRT-PCR kit (Quanta Biosciences Inc. Gaithersburg, MD) was used to reverse-transcribe and amplify each sample. Reverse transcription was carried out at 50°C for 30 minutes, followed by a PCR activation step at 95°C for 15 minutes, followed by amplification by 40 cycles of 1 minute at 94°C, 1 minute at 55°C, and 1 minute at 72°C, and a final extension of 10 minutes at 72°C. Each reaction was performed using a volume of 25 μL and contained 12.5 μL of reaction mix, 8.5 μL of RNA sample at 50 ng/μL, 1.5 μL (22.5 mM) of each forward and reverse primer, 0.5 μL of reverse transcriptase, and 0.5 μL (175 nM) of probe. Each sample was analysed in triplicate for each gene, and in every assay, one negative control and one reference sample were included for each gene. Each sample was amplified for 40 cycles, and at each cycle, the amount of fluorescence was quantified using a miniOpticon (Bio-Rad, Hercules, CA), and the cycle at which the signal rose above a fixed threshold (Ct) was determined. The products of the qRT-PCR reactions were also run on 1% agarose gels to ensure that only one band was present.

**Table 1:** Primer and probe sequences used in qRT-PCR

Gene	Forward Primer	Reverse Primer	Probe (contains fluorophore, 6-FAM™, on the 5' end and quencher, BHQ-1™, at the 3' end)
PAPPA2	GGGACAAGGAAGCTCTCAGT	CAGGGATCATCACAGGATTC	CATGCTTGGCCACACCAACATCATGATCCA
IGFBP-5	AAAGAGCTACGGCGAGCAAA	AGTAGGTCTCTTCAGCCATCTC	AGACTCTCGGGAACACGAGGAACCCA
PAPPA	CACAATGGACTCTGTGATGCT	TCTCCCTTCTAGGCAAAGGT	TGGTTCCCACCCATCGATGG
β-actin	CGTGAAAAGATGACCCAGAT	GGTACGACCAGAGGCATACA	ACCTTCAACACCCCAGCCATGT

### Protein extraction and Western blotting

Placentae and embryos were homogenized in 2 mL of T-PER™ Tissue Protein Extraction Reagent (PIERCE, Rockford, IL), incubated on ice for 7 minutes and then centrifuged at 10000 g for 5 minutes at 4°C. Complete protease inhibitor cocktail (Roche Applied Sciences) was added to the supernatant, which was then stored at -20°C.

Placental and embryonic samples containing 40 μg of protein were mixed with 5× SDS loading buffer and heated at 100°C for 10 minutes. 5 μL of PageRuler™ (#SM1811 Fermentas, Burlington, ON) was used as the molecular weight marker for each gel. Samples were run through a 4% stacking and 8% separating polyacrylamide gel for 60 minutes. Gels were run in duplicate, with one gel used to visualize total protein by staining with EZBlue™ (Sigma Aldrich, St. Louis, MO) for one hour at room temperature, followed by soaking in de-ionized, distilled water overnight to remove excess stain. The other gel was equilibrated in transfer buffer for 15 minutes and transferred onto a pure nitrocellulose membrane (Bio-Rad, Hercules, CA) using the Trans-Blot^® ^semi-dry electrophoretic transfer cell (Bio-Rad, Hercules, CA). After the transfer was complete, membranes were blocked for one hour at room temperature in Odyssey Blocking Buffer (Li-Cor Biosciences, Lincoln, Nebraska), incubated for one hour at room temperature in a solution containing 1:500 monoclonal mouse anti-actin (CLT9001; Cedarlane, Burlington ON) and 1:500 polyclonal goat-anti-human PAPPA2 antibody (AF1668; R&D Systems, Minneapolis, MN) diluted in Odyssey Blocking Buffer (Li-Cor Biosciences, Lincoln, NE) and 0.1% Tween-20 (Sigma, ON, Canada), washed 4 times for 5 minutes each in filter-sterilized PBS containing 0.1% Tween-20 at room temperature, and incubated in a solution containing 1:10000 fluorescently-labelled IRDye 800 anti-goat and IRDye 680 anti-mouse secondary antibodies (Li-Cor Biosciences, Lincoln, NE) diluted in Odyssey Blocking Buffer, 0.1% Tween-20 and 0.1% SDS for 45 minutes in the dark with gentle shaking. We performed all subsequent steps in the dark to avoid breakdown of the fluorescent dye. The membranes were again washed 4 times for 5 minutes each in filter-sterilized PBS containing 0.1% Tween-20 at room temperature, and rinsed with filtered PBS. Both total protein gels and anti-PAPPA2 blotted nitrocellulose membranes were visualized using the Odyssey infrared imaging system (Li-Cor Biosciences, Lincoln, NE). The two secondary dyes fluoresce at different wavelengths (anti-goat at 800 nm and anti-mouse at 700 nm), so we were able to simultaneously quantify the intensity of the PAPPA2 band (at approximately 250 kDa) and actin band (at approximately 40 kDa) on the same nitrocellulose membrane using the Odyssey software. Although the anti-PAPPA2 antibody we used was raised against human PAPPA2, previous Western blotting of different murine tissues with this antibody yielded results that correlated with mRNA levels measured by qRT-PCR using primers and a probe designed to target the mouse gene [23]. Furthermore, a negative control Western blot in which the primary antibody was omitted produced no bands (data not shown).

### Statistical analyses

We used the method of Pfaffl [29] to calculate mRNA expression levels for PAPPA2, PAPPA and IGFBP5, relative to the reference sample described above, e.g., a value of 1.5 indicates a sample has 50% more of a particular transcript than the reference sample, correcting for β-actin. To standardize PAPPA2 protein levels to actin, we divided the intensity of the PAPPA2 band by the intensity of the actin band, and then divided all of these ratios by the mean ratio of all samples (to yield a mean of 1 for all samples). RT-PCR and Western blotting data were analysed by ANOVA using JMP, Version 7 (SAS Institute Inc., Cary, NC).

## Results

### Placental gene expression

**Figure 1:**
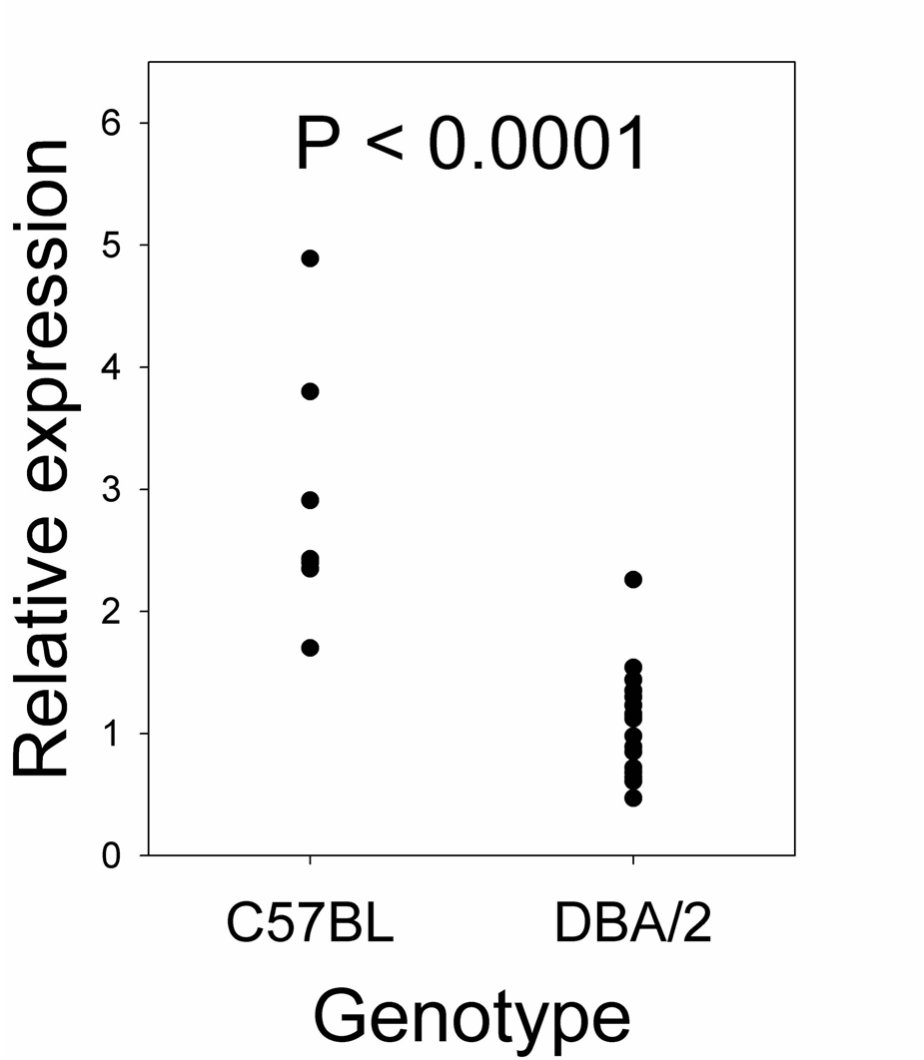
**Effect of QTL genotype on relative expression levels of placental PAPPA2 mRNA at day 12.5 post-coitum**. Quantitative RT-PCR was used to measure levels of PAPPA2 mRNA in mice homozygous for the C57BL/6 (N = 9) or DBA/2 (N = 17) QTL genotype, and a significant difference between genotypes was observed (F_(1,24) _= 39.8, P < 0.0001). Expression levels are relative to those of the reference sample and are standardized to β-actin.

**Figure 2:**
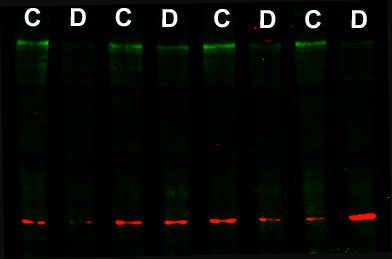
**Effect of QTL genotype on placental PAPPA2 protein expression at day 12.5 post-coitum**. Western blot of 8 representative samples of C57BL/6 (C) and DBA/2 (D) genotype placentae. The nitrocellulose membrane was scanned for fluorescence at 700 and 800 nm simultaneously. Fluorescence at 700 nm is shown in red and corresponds to actin (approximately 40 kDa) whereas fluorescence at 800 nm is shown in green and corresponds to PAPPA2 (approximately 250 kDa).

We examined the expression of PAPPA2, its substrate (IGFBP5) and a closely related gene (PAPPA) at 12.5 days post-coitum in mice that differ in genotype at the 1 MB QTL region, but are otherwise genetically homogeneous. The mRNA levels of β-actin in the placenta did not differ between genotypes (F_(1,23) _= 0.12, P = 0.73), indicating that this housekeeping gene was suitable for standardizing samples. There was a significant effect of genotype on placental PAPPA2 mRNA, with average PAPPA2 mRNA levels in C57BL/6 genotype placentae approximately 2.5-fold higher compared with DBA/2 genotype placentae (F_(1,24) _= 39.8, P < 0.0001; Table 2, Fig. 1). This difference was also significant when PAPPA2 mRNA levels were not standardized using β-actin (data not shown). Western blotting of placental samples with anti-PAPPA2 showed a primary band of approximately 250 kDa, consistent with previous work [23]; smaller bands may represent degraded fragments of PAPPA2. Placental levels of PAPPA2 protein differed between genotypes (F_(1,18) _= 12.0, P = 0.003; Table 2, Fig. 2), and the difference was similar in magnitude to that for placental mRNA expression. The difference at the protein level was significant whether or not PAPPA2 protein levels were standardized using quantification of actin (data not shown). Actin levels quantified by Western blotting did not differ between genotypes (F_(1,18) _= 0.16, P = 0.69). Including female age as a covariate in the analyses of the effect of genotype did not affect the results (data not shown). The placental mRNA levels of PAPPA and IGFBP-5 did not differ between genotypes (PAPPA: F_(1,24) _= 0.56, P = 0.46; IGFBP-5: F_(1,24) _= 0.08, P = 0.78; Table 2). We were not able to detect IGFBP-5 protein in the placenta by Western blotting.

**Table 2:** Effects of QTL genotype on placental and embryonic gene expression and weight, birth weight and litter size

	C57BL/6	DBA/2	P-value
*Placental gene expression*	N = 9	N = 17	
PAPPA2 mRNA	2.8 ± 0.2	1.1 ± 0.2	< 0.0001
PAPPA2 protein^a^	1.5 ± 0.2	0.6 ± 0.2	0.003
PAPPA mRNA	2.3 ± 0.6	1.8 ± 0.5	0.46
IGFBP-5 mRNA	1.5 ± 0.4	1.6 ± 0.3	0.78
*Embryonic gene expression*	N = 9	N = 17	
Embryonic PAPPA2 mRNA	0.8 ± 0.1	0.8 ± 0.1	0.69
*Phenotypic outcomes*	N = 10	N = 16	
Placental mass at E12.5 (g)	0.084 ± 0.007	0.087 ± 0.005	0.72
Embryonic mass at E12.5 (g)	0.091 ± 0.004	0.083 ± 0.003	0.16
Number of embryos at E12.5	3.8 ± 0.5	4.1 ± 0.4	0.71
Number of spontaneous abortions at E12.5	1.5 ± 0.3	0.9 ± 0.3	0.21
Birth weight (g)^b^	1.38 ± 0.03	1.31 ± 0.04	0.32
Litter size^b^	3.2 ± 0.5	3.8 ± 0.7	0.55

mRNA and protein levels are expressed relative to reference samples run in each assay

Values are least squares means ± standard error

^a^Sample sizes were reduced for PAPPA2 protein measurements (to 9 C57BL/6 and 11 DBA/2) because some females carried only one embryo and placenta, which were used for mRNA quantification

^b^Birth weight and litter size were measured in 58 C57BL/6 genotype pups from 13 females and in 30 DBA/2 pups from 8 females

### Embryonic gene expression

As in the placentae, embryonic mRNA levels of β-actin did not differ between genotypes (F_(1,19) _= 0.29, P = 0.60) and so this gene was used for standardizing samples. We did not detect a significant difference between genotypes in embryonic PAPPA2 mRNA levels (F_(1,25) _= 0.16, P = 0.69). Levels of PAPPA2 mRNA were much lower in the embryo (Ct values 21-29) than in the placenta (Ct values 18-25), and PAPPA2 protein could not be consistently detected in embryos by Western blotting.

### Phenotypic outcomes

There was no significant effect of genotype on embryonic (F_(1,25) _= 2.08, P = 0.16; Table 2) or placental (F_(1,24) _= 0.13, P = 0.72; Table 2) weight at E12.5 or the numbers of embryos (F_(1,25) _= 0.15, P = 0.71; Table 2) and spontaneous abortions (F_(1,25) _= 1.67, P = 0.21; Table 2).

Our line of mice breeds poorly, and of 33 females mated, only 17 produced live pups within two months of mating. The proportion of females that produced pups within two months did not differ between genotypes; 11 of 23 (48%) C57BL/6 genotype females produced pups, whereas 6 of 10 (60%) DBA/2 genotype females produced pups (χ^2^_1 _= 0.41; P = 0.52). There were additional matings that were not allowed to proceed for 2 months, in which some females produced pups, and since females from these matings are not included in the above calculations, these sample sizes are slightly different from those reported for newborn mass.

**Figure 3:**
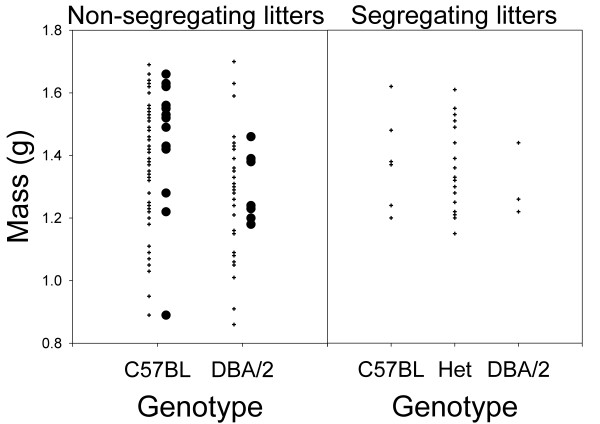
**Effect of QTL genotype on birth weight**. *Left panel: *Birth weight in litters that do not segregate for the QTL, produced by matings between homozygous parents. *Right panel: *Birth weight in litters that segregate for the QTL, produced by matings between heterozygous parents. Crosses represent individual pups, and circles represent the average litter mass per female. Average masses are not shown for segregating litters because they contain pups of more than one genotype.

There was no significant difference in birth weight between genotypes, regardless of whether we compared mean birth weight per female (F_(1,19) _= 1.30, P = 0.27), or performed a two-way ANOVA including genotype and female identity as factors, and used the mean sum of squares for female identity as the denominator when testing the effect of genotype (F_(1,19) _= 1.06, P = 0.32; Table 2, Fig. 3). Litter size at birth did not differ between genotypes (F_(1,19) _= 0.37, P = 0.55; Table 2), consistent with the lack of a difference in the number of embryos in females collected at embryonic day 12.5. We also measured birth weight in litters produced by heterozygous parents in which QTL genotype segregated within litters, and again found no effect of genotype (F_(2,18) _= 1.06, P = 0.37; Fig. 3).

We did not find any correlations between placental PAPPA2 mRNA or protein expression levels and placental mass (PAPPA2 mRNA r = 0.01, P = 0.97; PAPPA2 protein r = -0.29, P = 0.21) or embryonic mass (PAPPA2 mRNA r = 0.33, P = 0.10; PAPPA2 protein r = 0.28, P = 0.24).

## Discussion

In this study, we demonstrate that a QTL previously shown to affect postnatal growth in mice also affects the placental, but not embryonic, expression of PAPPA2. The placentae of C57BL/6 genotype mice exhibit a 2.5-fold higher expression of PAPPA2 mRNA than the placentae of DBA/2 genotype mice, and this difference is reflected in PAPPA2 protein expression. PAPPA2 presumably increases the local bioavailability of IGF-II via inactivation of IGFBP-5, although there are other mechanisms of IGFBP-5 action [22]. We could not observe this directly since IGFBP-5 protein levels were too low to be detected by Western blotting. Since IGF-II promotes growth, we would expect that increased levels would result in the promotion of growth in the placenta and therefore increased birth weight. However, despite a significant difference in placental PAPPA2 protein expression, we did not find any significant difference between genotypes in placental or embryonic weight at E12.5, or birth weight. We also failed to detect any difference in the placental mRNA levels of a closely related gene, PAPPA, suggesting that the expression of the latter gene is not altered to compensate for differences in PAPPA2 expression. The mRNA levels of PAPPA2's substrate, IGFBP-5, were not altered in the placenta in response to changes in PAPPA2 expression, suggesting a lack of feedback in this pathway. In contrast, Nishizawa *et al*. [18] observed increased IGFBP-5 mRNA levels in association with increased PAPPA2 levels in pre-eclampsia. A potential explanation for this discrepancy could be that PAPPA2 mRNA expression responds to altered IGFBP-5 protein expression, but IGFBP-5 mRNA does not respond to altered PAPPA2 protein expression. We were not able to detect IGFBP-5 protein by Western blotting, but such data would have little effect on our conclusions; we would expect to observe lower IGFBP-5 protein levels in association with elevated PAPPA2 expression, but failure to detect a significant difference would not provide evidence that IGFBP-5 levels were not affected.

Four recent studies have shown that PAPPA2 levels are elevated in the serum and placentae of mothers with pre-eclampsia and HELLP [17-20]. The magnitude of the difference in PAPPA2 levels between normotensive and hypertensive pregnancies ranged from approximately 2.5-[20] to 3-fold [17-19]. We observed a similar degree of variation between genotypes in our study, and yet we did not observe any phenotypic effects that would suggest abnormal placental function. This suggests that the altered expression of PAPPA2 found in recent human studies is not the cause of the pregnancy complications. Instead, PAPPA2 may be upregulated to compensate for the abnormal placentation found in pre-eclamptic pregnancies, since its upregulation would be expected to promote placental growth. A previous study by Nishizawa *et al*. [18] demonstrated an inverse correlation between maternal serum PAPPA2 levels and birth weight and between maternal serum PAPPA2 levels and placental weight in a population including both normotensive and pre-eclamptic pregnancies; however, they did not find a correlation within groups.

Our observation that a substantial alteration in placental PAPPA2 mRNA and protein expression has no detectable effect on birth weight suggests redundancy within the IGF pathway. Previous studies using PAPPA knockouts have shown that the ablation of PAPPA function in mice results in a 40% decrease in birth weight compared to wild-type littermates [30]. However, PAPPA expression is low in the murine placenta [31,32], so the observed decrease in birth weight was likely due to the lack of PAPPA expression in the embryo. Our mouse model showed variation in expression of PAPPA2 in the placenta but not the embryo, allowing us to examine the importance of placental PAPPA2 expression independent of that in the embryo.

A disintegrin and metalloproteinase 12 (ADAM12) is another metalloproteinase which is involved in the IGF axis, is highly expressed in placental tissue [33], and cleaves both IGFBP-3 and IGFBP-5 [34,35]. As with PAPPA, abnormally low levels of ADAM12 in the maternal circulation in the first trimester are associated with pre-eclampsia and IUGR [12,36]. Conversely, very high ADAM12 levels in the first trimester are associated with high birth weight [12]. However, mice deficient in ADAM12 have decreased viability, but have no major morphological abnormalities and are otherwise normal [37], consistent with the view that there is redundancy within the IGFBP pathway.

Few studies have examined the expression of PAPPA2 in any tissue; four papers have found differential expression between placentae of normotensive and hypertensive pregnancy [17-20], one has examined the proteolytic activity of circulating PAPPA2 [16], and one has compared PAPPA2 expression in the placentae of mice and humans [23]. In the present study, we found a difference in placental but not embryonic expression of PAPPA2, suggesting that there are tissue-specific regulatory elements involved in the control of PAPPA2 expression. Further research should examine the factors that regulate PAPPA2 in the placenta, and why PAPPA2 is upregulated during pre-eclampsia. While disregulation of PAPPA2 may not cause pregnancy complications, the factors that control its expression and cause it to be upregulated in pre-eclampsia may play a causative role in placental pathology. Understanding the regulation of PAPPA2 will help us understand the molecular causes of pre-eclampsia and possibly other pregnancy complications such as IUGR.

## Conclusions

Despite a significant difference between genotypes in placental PAPPA2 expression similar in magnitude to the difference between pre-eclamptic and normal placentae previously reported, we observed no difference in embryonic or birth weight. Our results suggest that elevated PAPPA2 levels are a consequence, rather than a cause, of pregnancy complications.

## Abbreviations

ADAM12: A disintegrin and metalloproteinase 12; IGF: insulin-like growth factor; IGFBP: insulin-like growth factor binding protein; IUGR: intrauterine growth restriction; qRT-PCR: quantitative real-time polymerase chain reaction; QTL: quantitative trait locus; PAPPA: Pregnancy-associated plasma protein A; PAPPA2: pregnancy-associate plasma protein A2

## Competing interests

The authors declare that they have no competing interests.

## Authors' contributions

Both PKW and JKC designed the study, carried out the mouse work, performed the molecular studies, analysed the data and wrote the manuscript. All authors read and approved the final manuscript.
